# Integrating phenotype ontologies across multiple species

**DOI:** 10.1186/gb-2010-11-1-r2

**Published:** 2010-01-08

**Authors:** Christopher J Mungall, Georgios V Gkoutos, Cynthia L Smith, Melissa A Haendel, Suzanna E Lewis, Michael Ashburner

**Affiliations:** 1 Genome Dynamics Department, Lawrence Berkeley National Laboratory, Berkeley, CA 94720, USA; 2 Department of Genetics, University of Cambridge, Downing Street, Cambridge, CB2 3EH, UK; 3 The Jackson Laboratory, 600 Main Street, Bar Harbor, ME 04609, USA; 4 Zebrafish Information Network, University of Oregon, Eugene, OR 97403-5291, USA

## Abstract

Phenotype ontologies are typically constructed to serve the needs of a particular community, such as annotation of genotype-phenotype associations in mouse or human. Here we demonstrate how these ontologies can be improved through assignment of logical definitions using a core ontology of phenotypic qualities and multiple additional ontologies from the Open Biological Ontologies library. We also show how these logical definitions can be used for data integration when combined with a unified multi-species anatomy ontology.

## Abstract 2

A phenotypic ontology that can be used for the analysis of phenotype-genotype data across multiple species, paving the way for truly cross species translational research.

## Background

The completion of the Human Genome Project [1,2] has resulted in an increase in high-throughput systematic projects aimed at elucidating the molecular basis of human disease. Accurate, precise, and comparable phenotypic information is critical for gaining an in-depth understanding of the relationship between diseases and genes, as well as shedding light upon the influence of different environments on individual genotypes. Natural language free-text descriptions allow for maximum expressivity, but the results are difficult to compute over. Structured controlled vocabularies and ontologies provide an alternative means of recording phenotypes in a way that combines a large degree of expressivity with the benefits of computability. A number of different ontologies have been developed for describing phenotypes, and whilst this is a welcome improvement over free-text descriptions, one problem is that these ontologies are developed for use within a particular project or species, and are not mutually interoperable. This means that it is difficult or extremely difficult to combine genotype-phenotype data from multiple databases - for example, if we wanted to search a mouse or zebrafish database for genes associated with a particular set of phenotypes associated with a human disease, this would require mapping between the individual phenotype ontologies.

If we are to combine the results of a variety of phenotypic studies, then phenotypes need to be recorded in a structured systematic fashion. At the same time, the system must allow for a high degree of expressivity to capture the wide range of phenotypes observed across a variety of organisms and types of investigation. Here we propose a methodology that can be used to add value to existing phenotype ontologies by mapping them to a common reference framework based on existing standard ontologies. We implement this methodology for four active phenotype ontologies, focusing primarily on a phenotype ontology used for the mouse. Our results also cover phenotype ontologies used for human and worm, and some exploratory work on plant trait ontology to demonstrate the generic utility of the approach. We demonstrate how our approach assists with the ontology development cycle, and we show how the addition of a multi-species anatomical ontology can enable queries across species.

### Open biological ontologies

**Figure 1:**
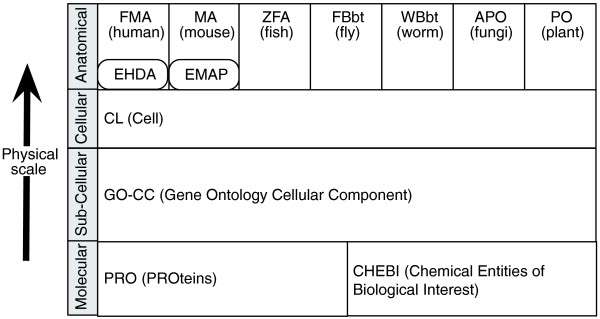
**OBO-registered ontologies of physical objects, from the molecular scale up to gross anatomical scale**. Above the cellular level, anatomical ontologies are partitioned taxonomically (the full breadth of taxonomic coverage in OBO is not shown). For mammals there is a second bipartite division, between fully formed structures and developing structures. The former are represented in the Foundational Model of Anatomy (FMA) and the adult Mouse Anatomy (MA), and the latter in the Edinburgh Human Developmental Anatomy (EHDA) and the Edinburgh Mouse Atlas Project (EMAP) ontologies.

Ontologies consist of collections of classes, arranged in a relational graph, to provide a computable representation of some domain. Examples of these domains include organismal anatomy, chemical entities, biological processes, phenotypes and diseases. The Open Biological Ontologies (OBO) project was created in 2001 as an umbrella body for the developers of life-sciences ontologies [3]. OBO was largely inspired by and grew out of the Gene Ontology (GO) Consortium. The GO [4] has been recognized as a key component in the integration of biological data, due in part to its wide use by disparate groups and its integration with other ontologies. One of the goals of OBO is to rationally partition the biological domain to minimize overlap between the ontologies, and to ensure logical coherence across ontologies, such that ontologies can be used in combination to describe complex biology. Figure 1 shows the OBO libraries partitioning of different kinds of physical objects, from whole-organism scale (anatomy) down to the molecular scale (chemicals and proteins). In this paper we focus on two broad categories of ontology: anatomical and chemical structural ontologies, and phenotype ontologies.

### Anatomical ontologies

There are a variety of ontologies representing anatomical entities such as hearts, brains and their parts. The current anatomical ontology space is segregated along taxonomic lines, with an anatomical ontology being maintained by each of the major multi-cellular model organism databases. In addition, there are anatomical ontologies for broader taxonomic groupings, such as teleost fishes and amphibians; these are focused on macroscopic anatomy and are used by evolutionary biologists [5,6]. Whilst this taxonomic division makes sense from an organizational perspective, the lack of a common ontology inhibits cross-species inferences (for example, finding zebrafish genes that are associated with phenotypes similar to those exhibited in a human disease). For the mouse, there are actually two ontologies - the mouse anatomy (MA) [7] and the Edinburgh Mouse Anatomy Project (EMAP) [8] ontologies, representing adult structures and developing structures, respectively. The situation is similar for humans, with adult human anatomy represented comprehensively in the Foundational Model of Anatomy (FMA) [9], and embryonic structures in the Edinburgh Human Developmental Atlas (EHDA). This division complicates queries even within a single species. The taxonomic partitioning of anatomical ontologies is largely at the gross anatomical level; cells and cellular components are represented in the OBO Cell ontology (CL) [10] and the GO cellular component ontology (GO-CC) and are applicable across multiple phyla. The decision to attempt to represent the full diversity of life across multiple phyla within these ontologies can complicate the development of the ontology, but the end result is more useful for cross-species queries. Similarly, the Common Anatomy Reference Ontology (CARO) [11] is an upper ontology for anatomy that consists of abstract structural classes that are extended by classes in individual anatomical ontologies in any taxon. This helps ensure that different anatomy ontologies are constructed consistently based upon common principles, but does not attempt to represent specific entities present in different species, such as hearts, blood, eyes, and so on. These anatomy ontologies are arranged as *is_a *hierarchies and often include additional relations such as *part_of *and *develops_from *[12].

### Molecular and chemical entity ontologies

Chemical Entities of Biological Interest (CHEBI) is an ontology of chemical entities [13]. The OBO Protein ontology (PRO) [14] is a classification of proteins and protein structures. At this time, PRO is a relatively new ontology, and many biologically important proteins are not yet represented. When combined with the anatomical ontologies mentioned above, we have broad coverage of physical entities at different levels of granularity, from the molecular scale up to the whole-organism level.

### Phenotype ontologies

Phenotype information has traditionally being captured using free-text fields in databases. Whilst this does allow for the full expressivity of natural language, the descriptions are largely opaque to computational inference. For example, if one curator uses the phrase 'increased size of jaw' and another uses the phrase 'mandible hyperplasia' to describe the phenotype associated with alleles of an orthologous gene in two different species, it is difficult for a computer to detect the similarity in these phenotypic descriptions without resorting to error-prone natural language processing techniques.

**Figure 2:**
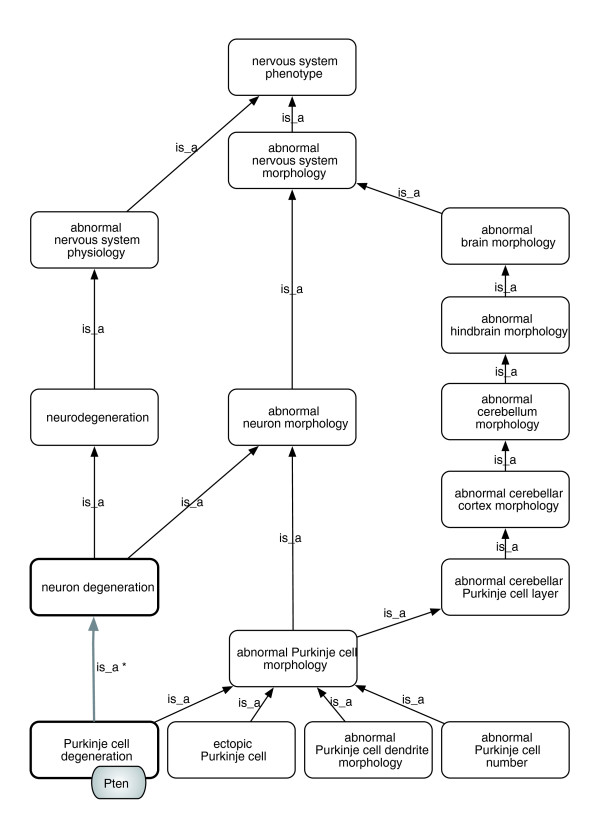
**Example portion of the MP, and the equivalence relations between MP classes and EQ descriptions**. Paths to the root over *is_a *links from 'Purkinje cell degeneration' and siblings. The *is_a *hierarchy is used for query-answering and genotype-phenotype analysis. Queries for 'neurodegeneration' or 'abnormal neuron morphology' should return genes or genotypes associated with 'Purkinje cell degeneration', such as the *Pten *gene. Note that prior to December 2008 MP lacked the highlighted link (indicated with the asterisk between two bold boxes), which resulted in false negatives for queries to 'neurodegeneration'. Using automated reasoning we were able to infer this link from the logical definitions and associated ontologies. We presented our results to the MP editors, who subsequently amended the ontology to include the link.

The success of GO has led several groups and communities to adopt or create phenotype ontologies using species-centric phenotype terminological standards. The structure of these ontologies, with classes arranged in an *is_a *hierarchy, allows for more intelligent searching and grouping together of genotypes and phenotypes within a species. For example, the database might record an association between a genotype of the mouse *Pten *gene and the class 'Purkinje cell degeneration' (MP:0005405); this genotype would be returned in a query for 'neurodegeneration' due to the graph structure and the transitivity of the *is_a *relation (Figure 2).

Examples of these species-centric phenotype ontologies include: the Mammalian Phenotype ontology (MP) [15]; the Worm Phenotype ontology (WP); the Plant Trait ontology (TO) [16]; the Human Phenotype ontology (HP) [17]; the Ascomycete Phenotype ontology (APO); and the Mouse Pathology ontology (MPATH). Whilst these ontologies serve their respective communities well, they are difficult to use for data integration across communities because there is no single ontology that is applicable to all species.

### PATO quality ontology and post-composed phenotype descriptions

Some model organisms, such as zebrafish and *Drosophila*, do not use species-centric phenotype ontologies but rather have opted for a compositional approach. That is, instead of choosing from predetermined lists of phenotypes, curators have the ability to compose descriptions of phenotypes on-the-fly using combination of classes from several ontologies, including an ontology of qualities termed Phenotype and Trait ontology (PATO) [18]. These composed descriptions minimally consist of at least two variables: the entity that is observed to be affected (for example, head, liver, Purkinje cell, and so on), and the specific characteristic or quality of that entity affected (for example, size, color, shape, structure). This is dubbed the 'EQ' model [19,20]. The E variable is filled with a class from any OBO ontology (for example, FMA, MA, EMAP or CL) and the Q variable is filled with a class from PATO. PATO covers both general qualities (for example, shape) and specific qualities (for example, branched), connected in a hierarchy of *is_a *relations. This EQ approach has been used in the annotation of human genotype-phenotype associations, as well as in model organism databases such as FlyBase (*Drosophila*) [21] and ZFIN (zebrafish) [22].

When phenotype descriptions are composed by the annotator at the time of annotation, we say that we are post-composing (or post-coordinating) the description. This is in contrast to the approach exemplified by the MP, in which descriptions are pre-composed (or pre-coordinated) in advance by the ontology editor. Table 1 shows the ontologies and methodologies currently used by various different projects. The pre- and post-composed approaches appear incompatible; it may seem that if we are to fully utilize model organism data for both translational and basic research, conformance to a single scheme may be a prerequisite. To the contrary, these differing methodologies and ontologies are complementary and fully compatible. We can still compute across species using these different approaches provided two criteria are met. First, there are equivalence statements between classes in pre-composed ontologies and PATO-based EQ descriptions. For example, the MP class 'small ears' can be declared equivalent to the EQ description composed from the PATO class 'small' and the mouse anatomy class 'ear'. This equivalence relationship constitutes a 'logical definition' for the phenotype class. Second, there is a means of linking across species-centric anatomical ontologies.

**Table 1:** Genotype-phenotype curation in different projects uses different ontologies and methodologies

Project	Organism	Methodology	Ontologies used	Entities annotated
MGI	Mouse	Pre-composed	MP	Genotypes
NIF	Mouse (neuro)	Post-composed	PATO, NIFSTD,	Organisms
WormBase	*Caenorhabditis elegans*	Both pre-composed and post-composed	WP	Genes
SGD	*Saccharomyces cerevisiae*	Pre-composed	APO	Genotypes
Gramene	Viridiplantae	Pre-composed	TO	Genotypes
FlyBase	*Drosophila melanogaster*	Post-composed	PATO, FBbt, GO	Genotypes, alleles
ZFIN	*Danio rerio *(Zebrafish)	Post-composed	PATO, ZFA	Genotypes
DictyBase	*Dictyostelium discoideum*	Post-composed	PATO, DDANAT	Genotypes
PATO OMIM-annotation project	*Homo sapiens*	Post-composed	PATO, FMA, CHEBI, CL, GO	Genotypes (corresponding to OMIM sub-records, for example OMIM:601653.0001)

We exclude annotation efforts that use free text in place of a publicly available ontology or terminology (such as the various genome-wide association study projects), or those not specifically focused on genotypic curation. NIF: Neuroscience Information Framework; DDANAT: Dictyostelium Discoideum Anatomy Ontology; FBbt: FlyBase anatomy ontology; MGI, Mouse Genome Informatics group at Jackson Laboratory; NIFSTD: Neuroscience Information Framework Standardized Ontology; SGD, Saccharomyces Genome Database; ZFA, Zebrafish Anatomy ontology; ZFIN, Zebrafish Information Network.

The lack of a set of equivalence mappings has hitherto been an obstacle to data integration across species using these different annotation approaches. In this paper we describe our methodology for connecting classes in pre-composed ontologies to EQ descriptions using an ontological framework - providing logical definitions for these classes. We illustrate this methodology primarily using the MP, and show that these mappings can be used to assist in ontology development through the use of automated reasoners. We also describe the construction of a multi-species anatomy ontology, which when combined with our EQ descriptions can be used to make cross-species queries.

## Results

### Formal representation of phenotypes

We logically define phenotypes by making an equivalence relation between classes in the pre-composed phenotype ontology to EQ descriptions, with each such description consisting of the following elements: Q, the type of quality (characteristic) that the genotype affects; E, the type of entity that bears the quality; E2, an additional optional entity type, for relational qualities; M, a modifier.

**Figure 3:**
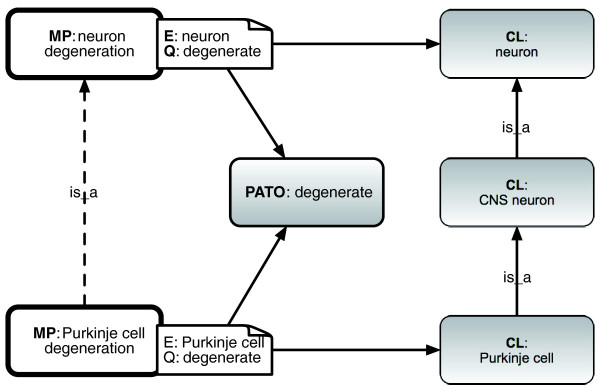
**Equivalence relations between MP classes and EQ descriptions**. Equivalence relations between two MP classes and their equivalent EQ descriptions. Here we treat MP 'degeneration' terms as in the PATO quality (Q) 'degenerate', rather than the process of degeneration. Here the bearer entities (E) are represented in the OBO Cell Ontology (CL). The EQ notation can be translated to logical expressions using Table 2. The dotted line indicates a relationship in the MP that can be independently inferred by a reasoner. CNS, central nervous system.

We can then translate the EQ description to an ontology language such as OBO Format or OWL (Web Ontology Language) - this allows us to use powerful general-purpose ontology tools such as automated reasoners to query and manipulate phenotype descriptions, and to compute subsumption hierarchies in phenotype ontologies (Figure 3). Ontology languages have a means of composing descriptions in a logically unambiguous fashion as intersections between classes. The modeling strategy used is described in detail elsewhere [23], but a brief summary as background follows here.

We use the formal *inheres_in *relation for relating qualities to their bearers. We treat the phenotype 'femur shape' as the class intersection of (a) the class 'shape' and (b) the class of all things that stand in an *inheres_in *relationship to a 'femur'.

In OBO Format this is written as:

intersection_of: PATO:0000052 ! shape

intersection_of: inheres_in MA:0001359 ! femur

Note that the text after the '!' is merely a comment, not a part of the format, used here to provide the human readable name for that class.

This can be read as a genus-differentia style definition, a <shape that inheres_in a femur>. We translate any EQ pair to <Q that inheres_in E>. For relational qualities we use the *towards *relation to connect the quality to the additional entity type on which the quality depends (for example, the concentration in urine of calcium). Here we use a simple 'EQ syntax' to explain our results, although the underlying representation is in OBO format (OBO Format, 2009). Table 2 shows the mapping between these two schemes. Our equivalence mappings are available in both OBO and OWL formats from the PATO wiki [24], or alternatively from the OBO logical definitions download page [25].

**Table 2:** Translation between variables in EQ templates and logic based OBO or OWL class intersections

EQ syntax	OBO syntax	OWL Manchester syntax
E = <E>	Intersection_of: <Q>	<Q> that inheres_in some <E>
Q = <Q>	Intersection_of: inheres_in <E>	
		
E = <E>	Intersection_of: <Q>	<Q> that inheres_in some <E>
Q = <Q>	Intersection_of: inheres_in <E>	and towards some <E2>
E2 = <E2>	Intersection_of: towards <E2>	
		
E = <E>	Intersection_of: <Q>	<Q> that inheres_in some <E>
Q = <Q>	Intersection_of: inheres_in <E>	and has_qualifier some <E2>
M = <M>	Intersection_of: has_qualifier <M>	

Phenotypes can be written using EQ syntax or as logical expressions in general purpose ontology languages such as OBO or OWL. Template variables are indicated by the angle brackets. For example, if <E> = 'femur' and <Q> = 'decreased diameter', then the OWL expression would be *decreased_diameter that inheres_in some femur*. Note that the qualifier relation is not yet in the Relations Ontology and is not formally defined, and is used as a placeholder for now.

We have developed a collection of equivalence mappings from classes in pre-composed phenotype ontologies to PATO-based formal description structures; we call these collections of mappings 'XP' ontologies (the 'XP' stands for cross-product). The descriptions are drawn from the cross-product of two sets of classes: the set of PATO classes and the set of classes from other OBO ontologies. For example, MP-XP is a collection of mappings between individual MP classes and their corresponding EQ descriptions. We can further partition the sets according to this scheme - for example, MP-XP-MA is the collection of such mappings whose descriptions are drawn from the cross-product of PATO classes and MA classes. Note that the mappings are all intended to be ones of equivalence - the EQ description should be neither more general nor more specific than the mapped pre-composed class.

In this paper we focus on the MP ontology. This is partly because of its relevance to translational research, maturity, comprehensiveness (6,844 classes), and to fulfill the data analysis needs of a particular project [20]. However, we also present preliminary results in mapping other pre-composed phenotype ontologies: HP, WP and TO. The last one was chosen to demonstrate the applicability of the technique outside metazoans. The mapping of the portion of HP corresponding to musculoskeletal phenotypes is described elsewhere [17].

The total number of classes, from MP, HP, WP and TO, for which we can map to PATO-based cross-product descriptions are summarized in Table 3. We attempt to achieve maximal coverage by combining initial automated term syntax parsing methods (see Materials and methods section), followed by manual curation of the results to check for biological validity. The MP-XP set has been curated most extensively, and of that set, the MP-XP-CL subset has been analyzed most thoroughly.

**Table 3:** Summary of equivalence mapping results

			Entity ontologies used				
			
Precomposed ontology	Total classes (non-obsolete)	Classes mapped using PATO	Gross anatomy ontology	CL	CHEBI	GO	MPATH
MP (mouse)	7,048	5,156 (73%)	3421 (MA)	738	294	1,064	194
			130 (EMAP)				
WP (worm)	6,341	1,177 (19%)	324 (WBbt)	32	114	570	
HP (human)	8,996	1,762 (20%)	1667 (FMA)	9	43	114	35
TO (plant)	958	398 (42%)	334 (PO)	2	106	2	

The number of classes in each pre-composed phenotype ontology is shown, together with the size of the subset of these classes that have been mapped to EQ descriptions. The EQ descriptions can be broken down further into subsets, depending on which ontologies are used. Note the subset numbers are not mutually exclusive, as there are scenarios where an EQ descriptions references multiple ontologies, so the numbers are not additive. PO, Plant Ontology (anatomical structure); WBbt, Worm anatomy ontology.

### Phenotypic mapping groups

The phenotype mappings fell into different overlapping categories, such as those based on basic anatomy, abnormality, compositional descriptions, processes, relational descriptions and absence. These phenotypes are described below, and Table 4 shows examples of these phenotype classes and the breakdown of their EQ description.

**Table 4:** Examples of equivalence mappings between pre-composed phenotype classes and EQ descriptions

Phenotype class	Bearer (E)	Quality (PATO)	Towards (E2)	Qualifier
**MP**				
Decreased diameter of femur	Femur	Decreased diameter		
MP:0008152	MA:0001359	PATO:0001715		
				
Spherocytosis	Erythrocyte	Spherical		
MP:0002812	CL:0000232	PATO:0001499		
				
Abnormal spleen iron level	Spleen	Concentration of	Iron	Abnormal
MP:0008739	MA:0000141	PATO:0000033	CHEBI:18248	PATO:0000460
				
Situs inversus	Visceral organ system	Inverted		
MP:0002766	MA:0000019	PATO:0000625		
				
Delayed kidney development	Kidney development	Delayed		
MP:0000528	GO:0001822	PATO:0000502		
				
Truncated notochord	TS20 notochord	Truncated		
MP:0004714	EMAP:4109	PATO:0000936		
				
Motor neuron degeneration	CL:0000100 motor neuron	Degenerate		
MP:0000938		PATO:0000639		
				
Axon degeneration	Axon	Degenerate		
MP:0005405	GO:0030424	PATO:0000639		
				
Loss of basal ganglion neurons	Basal ganglia	Has fewer parts of type	Neuron	
MP:0003242	MA:0000184	PATO:0002001	CL:0000540	
				
Abnormal Purkinje cell dendrite morphology	Dendrite of Purkinje cell	Morphology		Abnormal
MP:0008572	GO:0030425^part_of(CL:0000121)	PATO:0000051		PATO:0000460
				
**HP**				
Hypoplastic uterus	Uterus	Hypoplastic		
HP:0000013	FMA:17558	PATO:0000645		
				
Abnormality of vision	Visual perception	Quality		Abnormal
HP:0000504	GO:0007601	PATO:0000001		PATO:0000460
				
Narrow pelvis	Pelvis	Decreased width		
HP:0003275	FMA:9578	PATO:0000599		
				
**WP**				
Shruken intestine	Intestine	Shrunken		
WBPhenotype:0000086	WBbt:0005772	PATO:0000585		
				
**TO**				
Leaf area	Leaf	Area		
TO:0000540	PO:0009025	PATO:0001323		
				
Auxin sensitivity	Whole plant	Sensitivity	Auxin	
TO:000163	PO:0000003	PATO:0000085	CHEBI:22676	

Examples of pre-composed terms from four phenotype ontologies together with their logical definitions expressed as EQ expressions. The phenotype category can be seen by the ontologies used. Basic anatomical phenotypes use an anatomical ontology, unspecified abnormality can be seen in the final column. The one example of a compositional anatomical class (Purkinje cell dendrite is written as an OBO intersection expression. Processual phenotypes use the GO process ontology, and relational qualities have the E2 column filled in. PO, Plant Ontology (anatomical structure); WBbt, Worm anatomy ontology.

### Basic anatomical phenotypes

Most of the classes in the pre-composed phenotype ontologies are gross anatomy phenotypes - they can be defined in terms of a quality of some part of the body. For example: MP:decreased diameter of femur*; MP:hypothalamus hypoplasia; MP:large lymphoid organs; MP:muscular atrophy; MP:truncated notochord*; MP:motor neuron degeneration*; MP:axon degeneration*; HP:narrow pelvis*; TO:leaf area*; WP:shrunken intestine*; MP:situs inversus* (examples marked with an asterisk are shown in Table 4).

The first step to creating mappings for these pre-composed phenotypes is selection of the appropriate anatomical ontology. For worm and plant phenotypes, there is a single unified gross anatomy ontology covering each. For human phenotypes from HP, we use the FMA, and although the FMA does not include developing structures, this is not currently a limitation because the HP does not include many phenotypes for developing structures such as 'neural tube'.

The MP is intended as a mammalian phenotype ontology. Although most of the phenotypes defined are applicable to all mammals (and sometimes more general taxa) there is a bias towards mouse, as this ontology is generally used for mouse genotype annotation. This, and the fact that there was no general mammalian anatomy ontology, led us to use solely mouse anatomy (MA) ontologies for the decomposition of MP. We used MA (the adult mouse anatomy ontology) wherever possible. EMAP (Theiler stages 1 to 26) posed a problem due to the lack of generalized classes for developmental structures, such as 'notochord', forcing us to choose an arbitrary time stage-specific class (for example, 'notochord at TS20' to define 'truncated notochord'; Table 4). For cellular phenotypes such as 'motor neuron degeneration' we used CL, which is applicable across all taxa. For subcellular anatomy phenotypes, such as 'axon degeneration', we used the GO-CC ontology (also applicable across all taxa).

Many of the anatomical phenotypes are of the form 'abnormal X morphology' or 'increased/decreased size of X', where X is a class in the anatomy ontology or the cell ontology. Equivalence mappings for these were initially generated automatically (see Materials and methods). Manual assistance is required to map clinical terms such as 'situs inversus' (MP) to precise EQ descriptions (see Discussion).

The majority of all mapped phenotype classes fall into this category. This holds across all phenotype ontologies, but particularly for HP, which is by nature highly morphological.

### Abnormality

Both MP and HP are ontologies of abnormal phenotypes. Many classes are of the form 'abnormal X', where the exact nature of the abnormality is not specified; for example: MP:abnormal neuroepithelium of ampullary crest; MP:abnormal septation of the cloaca; HP:abnormality of vision*.

Here we elide a detailed discussion of what constitutes 'normal' or 'abnormal', as this is beyond the scope of this paper. We simply use a *has_qualifier *relation to replicate the intended structure of the MP class.

Note that the WP does not classify phenotypes as abnormal, but rather as 'variants'.

### Compositional descriptions of anatomical entities

Mapping a class such as abnormal Purkinje cell dendrite morphology* (MP:0008572) requires a slight variation on the basic EQ scheme. 'Purkinje cell' is represented in CL, and 'dendrite' is represented in GO-CC, but GO-CC does not specifically pre-compose 'Purkinje cell dendrite'. Logically, this presents no problem, as we can make an anonymous class defined using an intersection construct to specify this entity, using the *part_of *relation from the Relations Ontology. To accomplish this, we extended the simple EQ syntax such that we can use compositional expressions as IDs [26], and write the following:

E = dendrite^part_of(Purkinje_cell) Q = morphology M = abnormal

When translating the above EQ description to OBO or OWL we end up with a nested description, for example, in OWL Manchester syntax:

morphology that inheres_in some (dendrite that part_of some Purkinje cell) and has_qualifier some abnormal

However, tools that are downstream consumers of nested MP-XP class expressions must be able to interpret these appropriately, and the additional expressivity may pose problems for these tools. In addition, we need a way in which to present the descriptions in an intuitive manner to biologists.

We therefore extended EQ syntax to include the EW (Entity Whole) tag as below:

E = dendrite EW = Purkinje cell Q = morphology M = abnormal

This is equivalent to the above EQ description, but is simpler for tools to deal with, and simpler to present in tabular form to users.

This approach could be termed 'post-compositional', as the expression denoting the anatomical entity class is created after the anatomical entity ontology is deployed. However, the terminology becomes confusing here, so we reserve the term post-compositional specifically for the creation of such expressions at annotation time.

### Process oriented phenotypes

A significant number of classes in MP are described in terms of a biological process rather than a static description of an anatomical part. Examples include: MP:delayed kidney development*; MP:increased mast cell degranulation; TO:respiration rate; WP:hyperactive egg laying; HP:impaired spermatogenesis.

For these classes, we used PATO in combination with GO biological process (GO-BP) classes. PATO is divided at the top level between qualities of biological objects and qualities of processes. The former includes qualities such as size, shape, and structure and is used in conjunction with anatomical classes. The latter includes temporal qualities such as delayed, increased rate and is used in conjunction with GO-BP classes.

### Chemical entities and relational qualities

MP definitions occasionally reference types of chemical entities. For example: MP:hypocalciuria (excretion of abnormally low amounts of calcium in the urine); MP:abnormal spleen iron level*; TO:abscisic acid concentration.

Here we used the CHEBI ontology, typically using the CHEBI class as the related entity for a relational quality, where the bearer entity is a body substance such as blood or urine. In EQ syntax we would write the definition of hypocalciuria as:

E = urine Q = decreased concentration of E2 = calcium

For phenotypes that reference specific proteins such as 'interleukin-1' we can use the OBO PRO. At this time, the PRO does not include many of the required classes but these are easily added to the MP-XP definitions when they become available.

### Absence or change in number of parts

Mutations in or deletions of genes may result in the loss of a body part, or a change in the number of parts. Some example phenotypes are: MP:absent middle ear ossicles; MP:loss of basal ganglia neurons*; MP:alopecia (loss of hair); MP:absent spleen; WP:no oocytes; HP:polydactyly.

With PATO we typically describe absence in terms of the entity that is missing the part. For example, the following is problematic:

Q = absent E = spleen

Logically this is incoherent because there is no spleen to possess the quality of non-existence. Instead we can use a cognate 'relational quality' in order to compose a description:

E = abdomen Q = lacking all parts of type E2 = spleen

This second form is both more coherent and more expressive. For example, in defining 'loss of basal ganglia neurons' we can say:

E = basal ganglion Q = has fewer parts of type E2 = neuron

This obviates the need for a class 'basal ganglion neuron' (not present in the mouse anatomy ontology or the cell ontology). These PATO classes are grouped under the PATO class 'has number of' and have logical definitions that can be used in reasoning.

When translating 'absence' phenotypes to representations in ontology language such as OBO or OWL we have the option of treating the above description as a logical construct called a cardinality restriction. In OWL Manchester Syntax the absent spleen phenotype could be written as:

Abdomen that has_part exactly 0 spleen

This works for stating a number or number range, but cannot be used to state a relative increase or decrease in number. Another issue with the explicit representation is that it can create inconsistencies if it contradicts what is stated in the anatomy ontology. A full discussion is outside the scope of this paper, but one solution that has been previously proposed is to use non-monotonic logic [27].

### Validation using automated reasoners

A reasoner can be used to automatically classify (that is, place terms in the *is_a *hierarchy) a compositional ontology, such as a pre-composed phenotype ontology. We can also reverse the direction of implication, and use reasoners to validate the XP mappings based on the existing asserted *is_a *links in these ontologies. We used a variety of reasoning strategies to validate the MP mappings to EQs.

For each pre-composed phenotype ontology, we reasoned over the combined set consisting of the phenotype ontology, the XP mappings, and the ontologies referenced in those mappings. This yielded additional *is_a *links in the phenotype ontology, which were submitted to the maintainers of the ontology for approval, and often resulted in improvements to the ontology. For example, the reasoner suggested 'Purkinje cell degeneration' *is_a *'neuron degeneration' (inferred from the CL *is_a *hierarchy), which was previously missing from MP, and was promptly added [28]. In other cases the reasoner suggestions were rejected, because of problems in either the XP mappings or the referenced ontologies.

To validate this approach, we examined a particular subset, MP-XP-CL, the terms in MP for which there are mappings that involve CL. Using the OBO-Edit reasoner we inferred the existence of 88 possibly missing *is_a *relationships in MP. These were submitted to the MP curator for review. Of these, 48 were deemed to be correct, and the new links were added to the MP graph. One link was only partially correct, and resulted in a small rearrangement of a portion of the MP graph. Twenty-two links were rejected outright, and traced back to errors in the MP-XP-CL mappings, which were subsequently fixed. The remaining 17 are still pending, and mostly derive from inconsistencies between classification of normal cells in CL and abnormal cells in MP.

We also performed a partial validation of the mappings by attempting to recapitulate *is_a *links asserted in existing phenotype ontologies. We started by removing all *is_a *links from the phenotype ontology (but not from the ontologies referenced in the mappings) and attempted to recover these links using a reasoner. We found that 37% of the existing links in MP and 14% of the links in HP can be automatically reconstructed (Table 5). Of the false negatives (relationships between mapped classes that we cannot reconstruct), the problem was often an absence of supporting links in the referenced ontologies. For example, MP contains the statement 'asymmetric snout' *is_a *'abnormal facial morphology'. At the time of reasoning, the MA contained no relationships linking the classes 'face' and 'snout', which means there is no way to infer the stated MP link from first principles. After discussion, the MA curator (TF Hayamizu, personal communication) added a *part_of *link to the ontology between 'snout' and 'face', which was sufficient to allow inference of the MP link from the logical definitions. This is an example of how the combination of composing logical descriptions and using a reasoner can contribute to the development of a suite of ontologies, enforcing more consistency with one another. This is a guiding principle of the OBO Foundry. Table 5 also lists the novel relationships inferred by the reasoner; not all have been evaluated, and some will be true positives that will result in additions to the MP, such as the previously mentioned Purkinje cell example.

**Table 5:** Reasoner-inferred links for both human and mouse

	HP (human)	MP (mouse)
Number of *is_a *relationships asserted in ontology	10,162	7,950
Number of *is_a *relationships that can be inferred automatically	1,421	2,922
Number of novel *is_a *relationships proposed (unvetted)	407	478

To validate our approach, we attempted to derive existing non-redundant relationships in two phenotype ontologies based on equivalence mappings and external ontologies. The first row is the number of relationships manually asserted by the ontology editors. The second row is the number of these asserted relationships that we can independently infer from first principles. The final row is the number of novel new relationships found by the reasoner - some of these will be false positives, but others will represent genuine missing links in the ontology. A higher proportion was yielded for mouse due to the higher number of mappings (Table 3; we only expect to recapitulate relationships when we have mappings).

One problem we encountered was that the size of the combined ontologies proved too much for existing memory-bound reasoners to handle. We used two strategies to overcome this: using a relational database backed reasoner, which is not memory bound [29]; and ontology segmentation - dividing the reasoned set into manageable subsets. For example, rather than reasoning over all the ontologies referenced in MP-XP, we would select individual pair-wise subsets, such as MP-XP-MA, and reason over these sequentially. Both approaches have strengths and drawbacks; the relational database approach is too slow to be part of the ontology development cycle, and the simple pair-wise strategy can give incomplete results for complex phenotypes involving classes from more than one other ontology.

### A multi-species anatomy ontology for translational research

Our results show how classes in phenotype ontologies can be mapped to logical descriptions utilizing species-centric anatomical ontologies plus PATO qualities. These mappings enable us to query a mouse dataset, annotated using MP IDs such as MP:0001314 (corneal opacity), using the MA class 'cornea'. However, if we wish to query across combined multi-species datasets for all morphological phenotypes of the cornea, we need a more generalized class representing that which is shared by all vertebrate corneas. We have commenced construction of such a multi-species anatomical ontology, called Uber-ontology or Uberon. The current version of Uberon consists of over 2,800 classes, and it also contains links to over 9,300 classes in external, mostly species-centric anatomical ontologies. We do not attempt to generalize beyond metazoans [30]. Uberon is available from the main OBO website [31].

## Discussion

### Completion of the mappings

At the time of writing, MP-XP had the most comprehensive set of mappings (Table 3). The coverage of human phenotypes in HP-XP is poor by comparison for a number of reasons. The HP ontology is newer, and in comparison with MP, contains finer-grained morphological detail (exemplified by classes such as 'Bracket epiphyses of the middle phalanx of the 5^th ^finger', in which 'bracket' denotes a complex morphological phenomenon involving translocation along a radial-ulnar axis. We have recently started working with the editors of the HP ontology to extend PATO with the required morphological qualities and have proposed logical definitions for a further 1,000 classes that we are verifying with the HP editors and the assistance of a clinical geneticist (Peter Robinson, personal communication). The limited number of equivalence mappings for WP and TO reflect the fact that we have thus far focused on organisms more closely related to humans, but we have started working with the developers of these ontologies and training them to make these mappings as part of the ontology development cycle (Jolene Fernandez and Pankaj Jaiswal, personal communication).

Even within the relatively comprehensive MP-XP set, 27% of classes remain without a logical definition. With many of these the lack is due to missing classes in one or more ontologies. For these we make requests for new classes on the relevant OBO tracker and intend to go back and make the XP sets more comprehensive. In particular, we expect higher coverage as PRO becomes more comprehensive. Other classes make reference to pathological anatomical entities, such as hamartomas, which are outside the scope of MP - for these we are exploring the use of the MPATH ontology. At this time we have no good solution for classes such as MP:anhedonia, which require a publicly available behavior ontology (the Mammalian Behavior Ontology was not available at the time of writing).

### Logical equivalence between pre-composition and post-composition

Model organism databases and sources of human genotype-phenotype data are divided as to whether they use a pre-composed ontology of phenotype classes (such as the MP) or post-compose descriptions at the time of curation using PATO and other OBO ontologies (Table 1). There are merits and drawbacks to both approaches. The post-composition approach affords a much higher degree of freedom, but this comes with the price of adding complexity to the curation process and the potential to introduce an additional source of curator inconsistency. For example, recently a curator was annotating a paper in which a mutant organism was observed to have its internal organs transposed across the left-right axis of symmetry. An informal poll (OBO-Phenotype, 2007) [32] revealed that different curators would annotate this differently; using different anatomy or PATO classes. A pre-composed ontology such as the MP leaves less room for cross-curator variability: there is a ready-made class 'situs inversus' (MP:0002766) with the text definition 'lateral transposition or mirroring of the viscera of the thorax and abdomen, sometimes incomplete, with all organs maintaining the normal relative position with respect to each other'. In addition, the term 'situs inversus' has been part of the medical lexicon for hundreds of years. This is an advantage of pre-composed ontologies. However, if a curator observes a more specific form of situs inversus (perhaps with certain specific organs inverted), they will have to either request a new class or make do with the more general class. Using a post-compositional approach in which descriptions are composed at the time of annotation gives curators freedom without introducing a bottleneck to the curation process.

Happily we can have the best of all possible worlds. MP-XP includes an equivalence relation between 'situs inversus' and E="visceral organ system" [MA:0000019] Q = 'inverted" [PATO:0000625]. This means that annotations can be converted back and forth automatically. In addition, curators employing PATO to post-compose classes can look-up MP and MP-XP to determine which E and Q variables to use. In fact a mixed approach based on the work outlined here has been adopted by large scale mouse phenotyping efforts such as EUMODIC [19].

### Reconciling static and process-oriented perspectives

Note that there is sometimes a fine line between a process-oriented description and one described in terms of anatomical parts. For example, 'abnormal tooth development' (MP:0000116) could be defined in terms of the anatomical entity 'tooth' and the quality 'morphology' rather than the GO process 'tooth development'. However, this violates our principle that the mappings are formal ones of strict equivalence, as opposed to near-equivalence. In fact, MP declares 'abnormal tooth morphology' as a separate class (MP:0002100).

**Figure 4:**
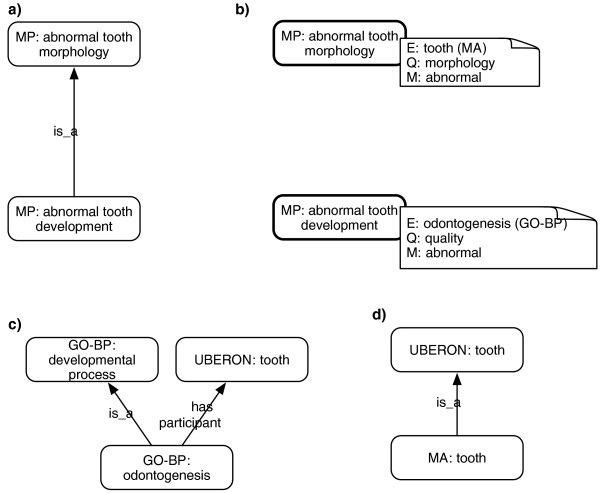
**Process and anatomical phenotypes**. **(a) **MP mixes process and anatomical/morphology phenotypes in the same *is_a *hierarchy. **(b) **MP-XP maps these to GO-BP and MA based descriptions respectively. **(c) **GO-BP to Uberon mappings make the link between the process class 'tooth development' and the general anatomical class 'tooth' explicit. **(d) **Uberon declaration stating that a mouse tooth is a subclass of the more general 'tooth' class.

Abnormal tooth development is not the same as abnormal tooth morphology, although they are correlated and presumably frequently observed together. In these situations we opted to make mappings to descriptions that corresponded exactly to the text definition in MP, using GO-BP classes if the phenotype class textual definition indicates a process phenotype. So we define 'abnormal tooth development' using GO and 'abnormal tooth morphology' using MA (Figure 4).

MP declares 'abnormal tooth development' to be a subtype of 'abnormal tooth morphology' (Figure 4a). The MP-XP mappings (Figure 4b) are insufficient to recapitulate this relationship automatically. We can add further mappings, such as GO-BP to Uberon [30] (Figure 4c) and the MA to Uberon mappings (Figure 4d). This is still insufficient to recapitulate the MP relationship using the axioms provided. However, it may be possible to generalize the logical definition of classes such as abnormal tooth morphology or to add logical rules to PATO such that it is possible to infer abnormal X morphology from abnormal X development using coordinated sets of ontologies. Or, alternatively, infer a new common subsuming phenotype such as 'abnormal tooth morphology or development'. This was outside the scope of the work described in the paper. We expect that using rules such as these will increase the number of relationships that can be recapitulated in pre-composed phenotype ontologies, and increase similarity scores between similar phenotypes that have been observed by different methods. For now we recommend curators follow principles of annotation laid down in [33] and annotate to both the process term and the anatomical structure term when indicated. For example, if it is known that the process of tooth mineralization was disrupted and that abnormal enamel morphology was observed, then curators should make two distinct annotations, one using the GO process class 'tooth mineralization' and another using an anatomical ontology class 'tooth mineral'.

### The challenges of coordinated ontology development

In this paper we have demonstrated how reasoners can be used to partially automate the placement of classes in phenotype ontologies. This requires making equivalence relationships between classes and logic-based description expressions. We note that it takes considerable effort to do this retrospectively rather than prospectively. Our approach here is retrospective - we take existing phenotype ontologies and then attempt to integrate them *post hoc*. Our preliminary work reveals that cryptic inconsistencies have evolved amongst ontologies that one would expect to be compatible (in that they all should conform to real-world biological knowledge); this will take some time and coordination to fix. For example, CL has 'pancreatic delta cell' as a subtype of 'enteroendocrine cell' but 'abnormal pancreatic delta cell morphology' and 'abnormal enteroendocrine cell' are unrelated in MP. In this case the MP hierarchy is correct, whereas the reference ontology in incorrect. These inconsistencies would continue to go unnoticed without explicit coordination.

Although it requires more of an initial effort to build in logical definitions (that is, assign EQ descriptions) from the outset (the prospective approach), we recommend this as a course of action for phenotype ontology development.

At the same time, whilst advocating this methodology, we recognize certain problems that need to be addressed. Describing phenotypes across a variety of scales and perspectives requires the use of a wide variety of ontologies. This requires that ontology developers become familiar with these ontologies, and that they coordinate more closely with the development of these ontologies. From a global OBO Foundry perspective this is a good thing, but it must be acknowledged that it requires additional effort from individual ontology developers. A more serious issue is that most reasoners do not scale to the combined union of ontologies within the OBO Foundry. More research on both improving reasoner scalability and ontology segmentation (that is, splitting the ontology into segments such as MP-XP-MA) is required.

### Anatomical ontology issues

In many cases we found that the MP was more detailed than the corresponding MA ontology. For example, the MP contains a class 'abnormal subarachnoid space morphology', but the MA does not contain the class 'subarachnoid space'. Our methodology here is to request classes from the MA editors and use these. Another acceptable approach would be to use ontologies specialized for a particular scientific field, such as the Neuroscience Informatics Framework (NIF) anatomical ontology (see [34] for brain phenotypes). The microscopic anatomical structures represented in the NIF-anatomy are, by design, applicable to both mouse and human; however, in this particular case the NIF-anatomy does not appear to contain the class that is needed. One might also consider using the FMA since it does contain a class 'arachnoid space' - however, we prefer not to mix and match classes from anatomical ontologies dedicated to different taxa as the differentia used for the logical definitions of a single pre-composed phenotype ontology (in this case MP), as this will be problematic for reasoning.

We also faced a problem defining classes such as 'truncated notochord'. MA only includes classes for the adult mouse. The EMAP ontology covers Theiler stages 1 to 26; however, EMAP was constructed according to different principles, with the result that there are no *is_a *relations and no single class 'notochord'. Rather, there are multiple such classes, one for each time stage and with no single general class abstracting over these stage-specific classes. There is also a new ontology EMAPA, which is an abstracted version of EMAP, but this still suffers from the same problem, with stage-specific classes and no *is_a *relations. Adopting CARO as an upper level ontology may address some of these issues.

The same dilemma arises with representing human anatomical entities (the FMA is for adult structures only), although currently most developmental phenotypes declared in the HP have a post-embryonic presentation.

### Uberon and translational research

We expect that perturbations in evolutionarily related genes and pathways across different species will give rise to similar phenotypes. This means that it should be possible to predict the phenotypic and clinical consequences of sequence variants based on genetic knowledge encoded in model organism databases. Previous studies have shown that these correlations can hold within a species for paralogous genes [35]. A major obstacle to extending this approach to orthologous genes is that phenotype data derived from multiple sources and species were semantically incompatible.

Now, by using a reasoner-backed database combined with the anatomical associations in Uberon and the mappings between the phenotype ontologies and respective EQ descriptions, we can ask questions and perform analyses in an automated fashion [20]. For example, given a phenotype such as 'corneal opacity' we can query across human, mouse and zebrafish annotations despite the heterogeneity of ontologies involved. This presents a major opportunity for transforming vital model organism data into knowledge of relevance to human health.

## Conclusions

We have provided a collection of equivalence mappings between classes in pre-composed phenotype ontologies and PATO-based EQ descriptions. Our mappings span four species. By translating EQ descriptions to logical axioms we used automated reasoners to validate our mappings, and demonstrated that many of the manually stated relationships in phenotype ontologies can be calculated automatically. This result indicates that logical definitions and automated reasoning can be used to make the ontology development cycle more efficient and consistent across ontologies.

We have also constructed an anatomical ontology that generalizes over existing metazoan species-centric ontologies. The combination of this ontology with our EQ mappings can be used to perform powerful translational cross-species queries and analyses of phenotypes recorded in separate databases using different ontologies. We believe that this will become a necessary and integral part of translational research involving genotype-phenotype associations.

## Materials and methods

In order to partially automate the generation of logical definitions, we defined an Obol [36] grammar that recapitulated the terminological syntax used in the different phenotype ontologies. For example, many MP class labels use a syntax that follows the simple grammar production rule:

phenotype → quality bearer

This yields a compositional description: <quality that inheres_in bearer>.

The terminal symbols in the grammar correspond to pre-composed classes in other ontologies. For example:

quality → (any PATO label or exact synonym)

bearer → (any OBO label or exact synonym)

For example "big ears' is translated to an obo genus-differentia definition 'increased_size that inheres_in ears'. In OBO format this is:

[Term]

id: MP:0000017 ! big ears

intersection_of: PATO:0000586 ! increased size

intersection_of: inheres_in MA:0000236 ! ear

The grammar is context-free, allowing us to have complex expressions describing the bearer; for example:

bearer → cell_component anatomical_structure

This yields a compositional description: <bearer that part_of bearer>

This allows us to parse the MP class "abnormal Purkinje cell dendrite morphology" as equivalent to the (nested) expression:

<PATO:morphology that inheres_in (GO:dendrite that part_of CL:Purkinje_cell)>

We can do this despite the absence of a pre-composed class 'Purkinje cell dendrite' in the GO cellular component hierarchy. The full set of grammars used can be seen at [37].

We employed a cyclical/iterative approach, with initial automatically generated cross-products manually inspected by two of us (GG and CJM) and fed into a curated cross-product ontology (MP-XP). The results were used to improve the grammar for subsequent runs. In addition, we used reasoners to check the logical entailments of the cross-product definitions. Sometimes this resulted in fixes to the pre-coordinated ontology; other times it revealed inconsistencies in our definitions. The entire process also resulted in numerous fixes to PATO and other OBO ontologies. Once we were confident in our definitions we engaged the editors of the phenotype ontologies more intensively to evaluate the cross-product definitions more thoroughly.

### Reasoning methods and tools

We tried a variety of reasoning tools, including OWL-based reasoners such as Pellet, FaCT++ and HermiT [38-40]. We also tried the OBO-Edit reasoner [41], the Obol reasoner and the OBD-SQL reasoner [42].

The only reasoner that could scale over the full set of ontologies plus mappings was the OBD-SQL reasoner, as it is the only reasoner that is not memory bound. For other reasoners we devised an ontology segmentation strategy involving reasoning over individual cross-product sets. For example, MP-XP-MA is the union of MP, MP-XP, PATO and MA. The results reported in this paper were obtained using the OBD-SQL reasoner. This reasoner works by initializing a relational database consisting of all asserted ontology relationships and then iteratively applying rules to derive new relationships until no new relationships can be derived.

## Abbreviations

APO: Ascomycete Phenotype ontology; CARO: Common Anatomy Reference Ontology; CHEBI: Chemical Entities of Biological Interest; CL: OBO Cell ontology; EMAP: Edinburgh Mouse Atlas (Theiler stages 1-26); FMA: Foundational Model of Anatomy (adult human anatomy ontology); GO: Gene Ontology; GO-BP, GO biological process; GO-CC: GO cellular component ontology; HP: Human Phenotype ontology; MA: Adult Mouse Anatomy Ontology, developed by the Mouse Genome Informatics group at Jackson Laboratory (Bar Harbor, Maine, USA); MP: Mammalian Phenotype ontology (sometimes MPO); MPATH: Mouse Pathology ontology; NIF: Neurosciences Informatics Framework; OBO: Open Biological Ontologies; OWL: Web Ontology Language; PATO: Phenotype and Trait ontology, an ontology of phenotypic qualities; PRO: Protein Ontology; WP: Worm Phenotype ontology (sometimes WBPhenotype); XP: cross-product (that is, equivalence mapping to a logical definition).

## Authors' contributions

CJM conceived of and coordinated the study, drafted the manuscript, created the initial mappings and performed the reasoner analysis. GG maintains mappings and coordinates changes with PATO. CS evaluated MP-XP for biological validity, evaluated reasoners results and coordinated changes with the MP. MAH and CJM conceived of and created Uberon. SEL and MA supervised the work and assisted with the manuscript.
